# Excessive Consumption of Alcoholic Beverages and Extremely High Levels of High-Density Lipoprotein Cholesterol (HALP) in the ELSA-Brasil Cohort Baseline

**DOI:** 10.3390/nu15051221

**Published:** 2023-02-28

**Authors:** Oscar Geovanny Enriquez-Martinez, Taísa Sabrina Silva Pereira, Jose Geraldo Mill, Maria de Jesus Mendes da Fonseca, Maria del Carmen Bisi Molina, Rosane Harter Griep

**Affiliations:** 1 Public Health Program, Health Sciences Center, Federal University of Espírito Santo, Vitória CEP 29047-105, Brazil; 2 Department of Health Sciences, University of the Americas Puebla, San Andrés Cholula 72810, Mexico; 3 Department of Physiological Sciences, Federal University of Espírito Santo, Vitória 29047-105, Brazil; 4 National School of Public Health, Oswaldo Cruz Foundation, Rio de Janeiro 21040-900, Brazil; 5 Postgraduate Program in Health and Nutrition, Federal University of Ouro Preto, Ouro Preto CEP 35400-000, Brazil; 6 Laboratory of Health and Environment Education, Oswaldo Cruz Institute, Oswaldo Cruz Foundation, Rio de Janeiro CEP 21040-900, Brazil

**Keywords:** alcohol, HDL-C, lipoprotein, cardiovascular, HALP

## Abstract

Background: It has already been established that the consumption of alcoholic beverages increases high-density lipoprotein cholesterol (HDL-C) levels in dose–response. Methods and Results:A cross-sectional analysis was carried out with 6132 participants of both sexes aged between 35 and 74 years, who were active and retired workers from six Brazilian states. Heavy drinkers were categorized by sex: men > 210 g/week and women > 140 g/week; moderate drinkers: men ≤ 209 g/week and women ≤ 139 g/week. The HDL-C level was dichotomized into normal (40 mg/dL–82.9 mg/dL) and extremely high (≥83 mg/dL). We used binary logistic regression to assess associations between baseline alcohol intake and HDL-C, which were adjusted for sex, age, income, physical activity, kilocalories and body mass index (BMI), and we found an positive association between extremely high HDL-C and the excessive consumption of alcoholic beverages. These participants were mostly women with a high income, lower waist circumference, kilocalorie consumption and also a higher consumption in all categories of alcoholic beverages. Conclusion: Excessive alcohol consumption was associated with a higher probability of extremely high HDL-C.

## 1. Introduction

The consumption of alcoholic beverages increased from 6.4 to 6.6 for the years 2016 to 2020 in per capita consumption in L, with estimates reaching 7.0 L for 2025 [1,2]; other estimates show that by 2030, the world per capita consumption will have reached 7.6 L, and the proportion of drinkers will increase by 0.22% annually, defined as a public health problem [3].

High-density lipoproteins (HDLs) have been considered as the ’good cholesterol’ that brings benefits to the body, mainly for cardiovascular health [4], through activity in the cholesterol efflux and anti-inflammatory, anti-oxidative, anti-thrombotic and anti-apoptotic characteristics [5,6,7].

Serum levels of HDL-C have been extensively investigated in relation to cardiovascular health. Low HDL-C levels have been associated with the incidence of cardiovascular diseases (CVDs) [8,9], leading to recommendations to increase this lipoprotein [10]. Currently, however, a U-shaped relationship is reported between HDL-C levels and cardiovascular events, showing that both low and very high levels of HDL-C presenta higher risk ofdeveloping cardiovascular events [11] and leading to mortality for all causes in both sexes [12].

Little evidence evaluating extremely high levels of HDL-C (HALP) in association with health, mainly cardiovascular disease, exists [13]. The main evidence describes genetic causes that generate HALP in Asian countries [14] and in the Dutch population [15]. In Latin America, there was one studyon the Brazilian population associated with increased carotid intima–media thickness [16], but studies on the association between HALP and CVD have not been concluded yet [17].

Contrary to what has already been reported regardingthe benefits of moderate consumption, the effects of excessive consumption areless clear and point to greater harmful effects on cardiovascular health, especially when evaluated amongbinge drinkers (episodic excessive drinkers) or with a specific type of alcoholic drink [18,19].

Our hypothesis is that the excessive consumption of alcoholic beverages increases HDL-C levels, reaching extreme levels of this lipoprotein. It has already been described that the excessive consumption of alcoholic beverages increases the risk of cardiovascular disease [20], and this behavior is identified as one of the biggest contributors to the burden of disease in the world increasing inflammatory and oxidative parameters that predispose one to a higher burden of CVD [1,21].

Some scientific evidence describes these relationships, e.g., the “Lipoprotein phenotyping study” reported an increase in the rate of cardiovascular events with HDL-C values >75 mg/dL [22]; in another study,“Incremental Decrease in End Point Through Aggressive Lipid Lowering” (IDEAL), these findings were reinforced with HDL-C >80 mg/dL [23].

We did not find any previous studies that directly relate excessive alcohol consumption and extremely high levels of HDL-C;it has only been described in a cross-sectional study of 3700 Russian subjects (75% men and 47% women)who hadexcessive consumption levels of alcohol [24]. Additionally, it is known that Russians have higher average levels of HDL-C cholesterol compared to populations in other countries; in addition to these levels, Russia stands out for having higher rates of cardiovascular disease when compared to other countries [25], suggesting a relationship in excessive alcohol consumption and HALP.

It is perceptible that the consumption of alcoholic beverages changes the health parameters of populations [26], and it certainly causesa modification in the lipid profile, which increases dose–response HDL-C levels [27]. The types of alcoholic beverages also play a role in modifying these parameters: positive associations have been found between wine consumption and increased HDL-C [28] and decreased HDL-C for beer [29].

This highlightsthe gaps in our knowledge of this association, especially with extremely high levels of HDL-C. Therefore, this study aimed to evaluate the consumption of excessive alcoholic beverages and itsrelationship with extremely high levels of HDL-C at the ELSA-Brasil baseline.

## 2. Materials and Methods

### 2.1. Study Design and Population

The current study is an observational, cross-sectional study developed from the baseline of the Longitudinal Study of Adult Health (ELSA-Brasil) [22]. The baseline of the ELSA-Brasil was established between 2008 to 2010 and consisted of data collection through interviews, examinations and laboratory analyses. ELSA-Brasil is a cohort of 15,105 adults: men and women who are active and retired workers from six higher education institutions (Federal University of Espírito Santo, Federal University of Minas Gerais, Federal University of Bahia, University of São Paulo, Federal University of Rio Grande do Sul and the Oswaldo Cruz-FIOCRUZ Foundation).

The ELSA-Brasil sample is made up of men and women aged 35–74 years who are eligible for the study; in terms of race/color,52% are white, 28% are brown or mixed color, 16% are black, 3% are Asian (mainly Japanese) and 1% are indigenous.Exclusion criteria for ELSA-Brasilarecurrent or recent (<4 months prior to the first interview) pregnancy, the intention to quit working at the institution in the near future, severe cognitive or communication impairment and, if retired, residence outside of a study center’s corresponding metropolitan area. ELSA-Brasil already has data from 3 waves: wave 1 (2008–2010), wave 2 (2012–2014) and wave 3 (2017–2018);ELSA-COVID (2020) and wave 4 (2022–2022) are currently being collected.

On a previously scheduled day, the participants appeared at 7:00 am in each research center for clinical, biochemical and questionnaire exams. In this way, a previously validated, comprehensive set of questionnaires, clinical measurements and laboratory tests wascarried out. As this wasa multicenter study, data collection was standardized.

Data werecollected in 2 phases. The first, lasting approximately1 h, included obtaining informed consent and conducting the initial interview at the participant’s job site. The second, comprising additional interviews and examinations, lasted for approximately 6 h and wasconducted at a study clinic.

**Figure 1:**
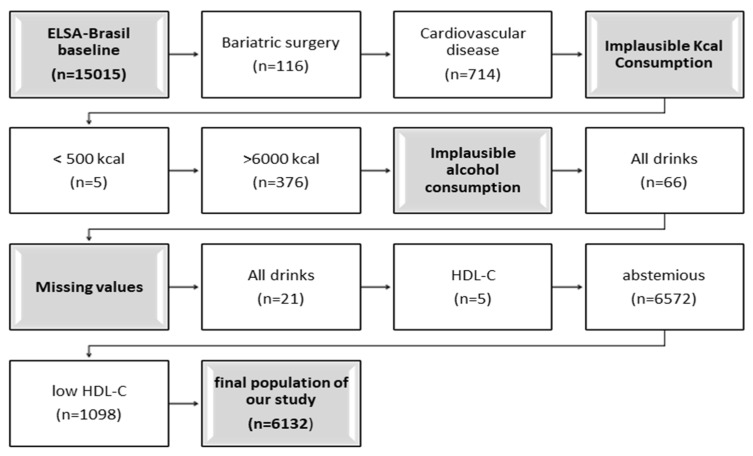
Exclusion criteria.

In this study, wave 1 was analyzed, formed by 6132 participants after we applied the following exclusion criteria: bariatric surgery, cardiovascular disease, implausible kcal consumption, implausible alcohol consumption, abstemious (0 mL/week), low HDL-C and missing data. (Figure 1).

### 2.2. Ethical Aspects

This study followed the international ethical standards found in the Declaration of Helsinki (2000). All procedures involving human subjects were approved by the Research and Ethics Committee of each country as follows:669/06 (USP), 343/06 (FIOCRUZ), 041/06 (UFES), 186/06 (UFMG), 194/06 (UFRGS) and 027/06 (UFBA). All participants signed a written informed consent form in both stages, with the anonymity of the information obtained being assured.

### 2.3. Study Variables

All covariates included in the analysis were obtained through face-to-face interviews by clinical or laboratory procedures, and the variables measured in this study were socio-demographic, lifestyle, anthropometric, diet, consumption of alcohol and biochemical (serum lipoproteins). The following socio-demographic variables were evaluated by closed questionnaires withvariables such asage (years), sex (male or female), income (in tertile), education level (incomplete primary, primary, high school or university), marital status (married, separated/divorced, single, widower or other—with previous union), ethnicity (not white or white). Lifestyle variables were evaluated using closed questionnaires or specific measures of lipid-lowering drugs (yes no no);body mass index (BMI) was used to classify nutritional status (thin <18.5 kg/m^2^; normal ≥18.5 kg/m^2^ and <24.9 kg/m^2^, overweight, obese ≥25 kg/m^2^) [25], and physical activity in leisure (low, moderate or high), smoking (never smoked, ex-smoker or smoker), consumption of alcoholic beverages (moderate or excessive), anthropometric measurements (weight (kg), height (mts) and waist circumference (cm)) were collected in a standardized way [30].Diet variables, (kilocalories, lipids, carbohydrates and proteins) were collected using the FFQ validated for the Brazilian population [31,32], as well as alcohol consumption (mL/week), beer (mL/week), spirits (ml/week), total alcohol (ml/week) and total alcohol (g/week)using the closed question questionnaire, and serum lipoproteins TC (mg/dL), triglycerides(mg/dL), HDL-C(mg/dL) and LDL-C (mg/dL)).

### 2.4. Alcohol Consumption

Alcohol consumption was reported through structured questionnaires with closed questions, which were used in each ELSA-Brasil research center to determine the types of alcoholic beverages (beer, wine and spirits), and the frequency and amount of consumption (daily, weekly or monthly) [33].

For this study, we worked with the total consumption of alcoholic beverages derived from the sum of millimeters/week of each participant and transformed it to g/week, classifying alcoholic beverage consumers as moderate and excessive drinkers according to the following equation:

Volume milliliter week alcohol × alcohol content/100 = Volume

Volume × 0.8 = grams/week

Heavy drinkers were categorized by sex: men >210 g/week and women >140 g/week; and moderate drinkers: men ≤209 g/week and women ≤139 g/week [34].

### 2.5. Blood Analysis

Blood samples were obtained by venipuncture using scalp and vacuum collection tubes. Fasting samples were collected, and at the time of collection, the participant was informed about the procedure, and we verified the fulfillment of the given guidelines through a questionnaire. The temperature of the collection room was maintained between 20 and 24 °C, and the samples were properly stored and transported to the project’s Central Laboratory, located at the University Hospital of São Paulo [35].

In this study, the biochemical parameters were obtained in two stages: after fasting for 8 to 12 h and 2 h after ingesting an overload of glucose. The samples were quickly processed to obtain serum, which was stored locally at −80 °C until it was sent to the ELSA-Brasil Central Laboratory (São Paulo) for the monthly determination of the analytes. The blood collection, processing, and biological transport protocol were standardized as described by Fedeli et al. [35]; the variables analyzed in this study were total cholesterol(enzymatic, colorimetric cholesterol oxidase method—ADVIA 1200 Siemens^®^), LDL-C (homogeneous colorimetric enzymatic method without precipitation), HDL-C (homogeneous colorimetric method without precipitation) and TGs (glycerolphosphate peroxidase method according to Trinder (enzymatic and colorimetric)). The tubes used for analysis, storage and transport were BD Vacutainer tubes with 9 mL volumes and BD Vacutainer scalps measuring 21 G and 23 G. For storage, a 2 mL Greiner Cryogenic Tube was used. The extremely high HDL-C cut-off point was definedby means of the 90th percentile, being, for this population, defined asHDL-C(≥83 mg/dL).Then, it was dichotomized into normal (40 mg/dL–82.9 mg/dL) and extremely high (≥83 mg/dL).

### 2.6. Statistical Analysis

The categorical (sociodemographic, lifestyle, anthropometric, consumption of alcohol and biochemical) variables dependent on HDL-C were analyzed using the chi-square test. The continuous (anthropometric, diet and biochemical) variables were expressed as mean and standard deviation and evaluated by the t-test. Likewise, they were dichotomized by HDL-C levels (normal and extremely high—HALP) and beverage consumption (moderate and excessive), performing the same statistical tests, depending on the nature of the variables.

Crude and adjusted binary logistic regression models were createdto estimate odds ratios (OR) and 95% confidence intervals (95% CI) to assess the association between HDL-C levels (dependent variable) and alcohol consumption (independent variable). The models used the dichotomized variables of HDL-C (normal and extremely high) and alcohol consumption (moderate and excessive). Model 1 was adjusted for sex, age and income, and model 2 was adjusted for model 1+ physical activity, kilocalories and BMI. All analyses were performed using Stata Statistical Software (release 13, StataCorp LP, College Station, TX, USA), and the level of significance was 5%.

## 3. Results

The study sample had 6132 participants, who were predominantly male (55.6%) and hada 6.8% prevalence of HALP. We analyzed socio-demographic, lifestyle, anthropometric, consumption and biochemical variables using the HDL-C levels. It was evident that most participants with HALP were female (*p* < 0.001), in the third income tertile (*p* < 0.001), completed higher education (*p* < 0.001), were married (*p* < 0.001), did not consume lipid-lowering drugs (*p* < 0.001), had a normal nutritional status (*p* < 0.001), had low leisure-time physical activity (*p* < 0.001) and had a moderate consumption of alcoholic beverages (*p* < 0.001). Table 1

The highest means for HALP were for age (*p* < 0.001), total cholesterol (*p* < 0.001) and HDL-C (*p* < 0.001) Table 2.

Table 3 shows the socio-demographic, lifestyle, anthropometric, consumption and biochemical variables dependent on HDL-C levels and alcohol consumption. When analyzing the consumption of alcoholic beverages, we found participants categorized with HALP in the two categories of consumption (moderate and excessive) were mostlywomen (*p* < 0.001), had a university degree (*p* = 0.005) and had a normal body mass index (*p* = 0.050). Higher proportions were observed in moderate consumption for those who had never smoked and in excessive consumption for ex-smokers (*p* < 0.001).

When analyzing the anthropometric, consumption, and biochemical variables depending on HDL-C levels and alcohol consumption, we found higher means in excessive consumption for weight (*p* = 0.023), height (*p* = 0.006) waist circumference (*p* = 0.011), kilocalorie consumption (*p* = 0.040) and all the categories of alcoholic beverages (*p* < 0.001) and for the consumption of wine (*p* = 0.007) (Table 4).

Table 5 presents the binary logistic regression with progressive adjustments (sex, age, income, physical activity, kilocalories and BMI). We found a positive association with extremely high levels of HDL-C (HALP) and excessive alcohol consumption (OR = 1.92; 95% CI (1.4–2.5)).

## 4. Discussion

The objective was to evaluate the excessive consumption of alcoholic beverages and the association with extremely high levels of HDL-C (HALP) in a Brazilian population; a positive relationship was found.

Participants with excessive drinking are more likely to have extremely high levels of HDL-C (HALP). These participants are mostly women with a high income, lower waist circumference, lower kilocalorie consumption and higher consumption in all categories of alcoholic beverages.

We found a prevalence of 11.8% of excessive alcohol consumption, which agrees with a population-based longitudinal study [29] and with the Brazilian population [36]. In our study, a prevalence of 6.8% of HALP was also estimated, which is lower in North Americans (3.8%) [37] and Asians (1.9%) [11], because the cut-off points for defining HALP in these populations were higher than in our study.

HDL-C levels differ when compared by sex, which is evident in our study, as the largest proportion of participants with HALP were women. The behavior of this lipoprotein and the corresponding changes depend on the physiological and hormonal statuses of women, showing an inverse association between the serum levels of HDL-C and estrogens [38]. Menopause is, therefore, a key factor in this relationship, as postmenopausal women have higher HDL-C values when compared to premenopausal women [39]. The physiological stage present in our population, determined by the average age of our sample, is consistent with the onset of these hormonal and physiological changes.

There is a direct relationship between education and cognition, relating to behaviors in favor of health care [40]. Consistent evidence shows this association in women [41,42,43], and the highest proportions of HALP were found in participants with high incomes and education levels, which is a result also evident in Chilean women [44]. It is important to recognize the nature of our sample, as they are employed or retired from federal universities, characteristics that impact sociodemographic variables in relation to the general population, especially those with higher education levels and incomes.

Our findings show a lower value of triglycerides in participants with HALP, agreeing with a study that evaluated sixNorth American cohorts, explaining this physiological behavior by abnormalities in lipid metabolic pathways, especially in the exchange of HDL-C to VLDL particles [45].

Another important factor is nutritional status: an imbalance in this variable is consideredas a risk factor for the global burden of disease [46]. In turn, obesity increases the risk of cardiovascular diseases, generating atheroma and changes in lipoproteins (dyslipidemias) [47]. The inverse association between nutritional status and serum HDL-C levels is well known. Increased BMI and WC present greater chances of low HLD-C, explaining this relationship in 57.1% and 36%of cases, respectively [48].

The guidelines which aim to reduce weight as a strategy to increase HDL-C [49,50] were found to be relevant in our study, as participants with HALP have lower levels of anthropometric variables. It is well known that the effect of weight loss by dietary modification, physical exercise or bariatric surgery influence changes in lipoproteins [51].

Different health outcomes are associated with the consumption of alcoholic beverages, depending on the quantity and type of these beverages, mainly related to cardiovascular effects [52], diabetes [53] and cancer [54], leading to mortality from all causes [55]. The findings relating the consumption of alcoholic beverages and HDL-C are emphatic in the relationship between these two variables, in dose response [27] or also in the form of “u”or “J”-shaped responses, attributing the protective role to HDL-C. These responses may result from activity on the efflux of cholesterol, and anti-inflammatory, antioxidant, antithrombotic and antiapoptotic characteristics [5,6,7], leading to a false hypothesis of cardiovascular protection with the association of alcoholic beverages and HDL-C levels [12]. However, this increase in HDL-C levels, dependent on the consumption of alcoholic beverages, must be carefully evaluated. Currently, there are some counterpoints to be analyzed when establishing these relationships. In a large population-based study in Japan, a genetic mutation was found in the cholesterol ester transport protein enzyme (CETP), responsible for the metabolism and levels of HDL-C. These findings report an association ofthe incidence of ischemic changes, being statistically significant and U-shaped, with very low and extremely high levels (HALP) of HDL-C [11].

Increased HDL-C levelshavebeen taken as evidence that therapeutic recommendations need to be made for the benefit of cardiovascular health;it is known that CETPis a mediator in the exchange of cholesterol ester and reverse cholesterol. For this purpose, specialized drugs have been developedfor the inhibition of CETP, demonstrating that when this protein is inhibited, HDL-C levels increase considerably, reaching extremely high levels (HALP) with a significant increase in mortality and cardiovascular events [56,57]; it has been failedto hypothesize that just increasing HDL-C at very high levels could be beneficial to cardiovascular health. This level of HDL-C may favor atherogenesis, because the molecules that predominate are HDL-2, rich in cholesterol esters and less able to carry out reverse cholesterol transport [58].

The antioxidant potential attributed to HDL-C in plasma has also been analyzed. There are other components with greater antioxidant potential than isolated HDL-C, such as fibrinogen, immunoglobulin G, uric acid albumin and ascorbate, with albumin being the dominant antioxidant and HDL-C with lower power 1–2% total antioxidant contribution in plasma [59], leading researchersto reflect on the antioxidant role of this lipoprotein.

The findings suggest that the relationship between HDL-C levels and cardiovascular health is still complex, going beyond just plasma concentrations [60]. HDL-C is a heterogeneous lipoprotein with different components, so the association with CVD should be further analyzed. HDL-C has been found to contain alpha-2 macroglobulin, CoC3 (complement C3), HP (haptoglobin) or PLMG (plasminogen), leading to the idea that not only will the increase in HDL-C contribute to cardiovascular protection, but also, the balance between the intrinsic components of these lipoproteins [61] and molecules that interact in HDL-C metabolism [23] should bothbeevaluated.

The effects of alcohol on health are still controversial, mainly due to itscharacteristics, referring to type, consumption pattern and quantity. It has already been described that excessive consumption has a negative impact on the health of the population and has implications for public health [1,62].

The possible mechanism by which the consumption of alcoholic beverages brings benefits to cardiovascular health is attributed to the increase in HDL-c levels, with a false belief that extremely high levels could bring even greater benefits [29].

In a recent meta-analysis, it wasshown that interest in studying the effects of alcohol consumption has increased considerably in recent years, in addition to reports of an increase in HDL-C levels (3.66 mg/dL 2.22–5.13) per 30 g of alcohol/day [63], associated with an increase in the rate of lipoprotein transport [64]. However, the results are still controversial, as the mechanisms by which alcohol influences HDL-C are not fully understood.

Our results should be considered in terms of clinical implications, as the increase in the dose–response of alcohol consumption and HDL-C levels should be analyzed carefully, as it would lead to the idea that excessive consumption would further increase HDL-C levels. C. It is known that extremely high levels of HDL-C are associated with dysfunctions in lipid metabolism or genetic load, reflected in non-functional HDL-C and correlated with greater risks in cardiovascular health.

Therefore, when an extremely high level of HDL-C is observed, it must be classified as a risk indicator, and health treatment must be given.As we currently think that HDL-C has no risk point, the recommendations only suggest increasing it without a cutoff point; this has clinical implications because the determination of HDL-C levels is a routine and easy test that brings important information that is sometimes undervalued.

The strengths and weaknesses of this study werethat ELSA-Brasil is not a representative study of the Brazilian population, but it represents an important portion of the population, providing important data for South American countries. The alcoholic beverage instrument has memory bias, but it was applied by trained personnel and had data quality control. Participants with implausible consumption were excluded.Thisis one of the first findings in a South American population, presentingimportant information about this population that has specific characteristics.

Finally, the consumption of alcoholic beverages in the world is increasing, and the mechanisms of association with cardiovascular health should be better elucidated. Few studies have tried to understand the specific effect of HDL-C, and this research is a pioneer in this association.

## 5. Conclusions

Excessive alcohol consumption was positively associated with extremely high HDL-C (HALP). The increase in serum levels caused by the consumption of alcoholic beverages must be carefully analyzed, because HDL-C is a heterogeneous lipoprotein, and high levels do not provide cardiovascular protection.

## Figures and Tables

**Table 1:** Socio demographic, lifestyle, anthropometric, consumption and biochemical variables dependent on HDL-C levels.

	Normal	HALP	*p*
	*n* (%)	*n* (%)	
	**5717 (93.2)**	**415 (6.8)**	
Sex			
Male	3312 (57.9)	100 (24.1)	<0.001
Female	2405 (42.1)	315 (75.9)	
Income Tertile			
1	1926 (33.7)	107 (25.8)	<0.001
2	1907 (33.4)	133 (32.1)	
3	1874 (32.8)	174 (42.0)	
Education Level			
Primary incomplete	236 (4.1)	20 (4.8)	0.038
Primary complete	305 (5.3)	18 (4.3)	
High school complete	1722 (30.1)	100 (24.1)	
University complete	3454 (60.4)	277 (66.7)	
Marital Status			
Married	3995 (69.9)	227 (54.7)	<0.001
Separated/divorced	910 (15.9)	78 (18.8)	
Single	467 (8.2)	68 (16.4)	
Widower	155 (2.7)	19 (4.6)	
Other (with previous union)	190 (3.3)	23 (5.5)	
Ethnicity			
Not white	2371 (42.0)	171 (41.7)	0.918
White	3269 (58.0)	239 (58.3)	
lipid-lowering drugs			
No	5037 (88.1)	390 (94.0)	<0.001
Yes	680 (11.9)	25 (6.0)	
Physical activity in leisure			
Low	4144 (73.6)	274 (66.2)	0.005
Moderate	872 (15.5)	84 (20.3)	
High	618 (11.0)	56 (13.5)	
Smoking			
Never smoked	2881 (50.4)	204 (49.2)	0.326
Ex-smoker	1882 (32.9)	150 (36.1)	
Smoker	953 (16.7)	61 (14.7)	
Consumption of alcoholic beverages			
Moderate	5041 (88.2)	352 (84.8)	0.043
Excessive	676 (11.8)	63 (15.2)	

Chi-square tests; data were expressed (%) for categorical variables. *p* < 0.05.

**Table 2:** Anthropometric, diet and biochemical variables dependent on HDL-C levels.

	Normal Median ± SD	HALP Median ± SD	*p*

*n* (%)	5717 (93.2)	415 (6.8)	
Age (years)	51.8 ± (8.9)	54.0 ± (8.7)	<0.001
BMI (Kg/m^2^)	26.7 ± (4.4)	24.5 ± (3.9)	<0.001
Waist circumference (cm)	91.8 ± (12.3)	82.7 ± (10.5)	<0.001
Kilocalories	2933.1 ± (997.8)	2671.4 ± (899.5)	<0.001
Lipids	94.3 ± (36.5)	86.4 ± (34.7)	<0.001
Carbohydrates	374.6 ± (144.6)	334.4 ± (125.5)	<0.001
Proteins	137.7 ± (53.1)	130.7 ± (52.8)	0.009
Wine (mL/week)	248.6 ± (418.1)	283.9 ± (424.7)	0.097
Beer (mL/week)	1380.8 ± (1736.7)	1259.6 ± (1740.0)	0.171
spirits (mL/week)	39.6 ± (116.6)	49.7 ± (117.1)	0.255
Total alcohol (mL/week)	1669.2 ± (1756.9)	1593.4 ± (1784.9)	0.397
Total alcohol (g/week)	91.5 ± (88.8)	93.1 ± (103.0)	0.722
TC (mg/dL)	218.9 ± (40.6)	241.4 ± (40.3)	<0.001
Triglycerides (mg/dL)	145.0 ± (109.7)	93.6 ± (67.8)	<0.001
HDL-C (mg/dL)	56.6 ± (10.6)	93.4 ± (11.3)	<0.001
LDL-C (mg/dL)	134.3 ± (34.4)	129.6 ± (36.3)	0.007

*t*-test. Data were expressed as mean ± SD for continuous variables. *p* < 0.05.

**Table 3:** Socio demographic, lifestyle, anthropometric, consumption and biochemical variables dependent on HDL-C levels and alcohol consumption.

	Normal			HALP		
	*n* (%)		*p*	*n* (%)		*p*
	**Moderate**	**Excessive**		**Moderate**	**Excessive**	
	**5041 (88.2)**	**676 (11.8)**		**352 (84.8)**	**63 (15.2)**	
Sex						
Male	2825 (56.0)	487 (72.0)	<0.001	70 (19.9)	30 (47.6)	<0.001
Female	2216 (44.0)	189 (28.0)		282 (80.1)	33 (52.4)	
Income Tertile						
1	1669 (33.2)	257 (38.0)	0.023	84 (23.9)	23 (36.5)	0.108
2	1707 (33.9)	200 (29.6)		115 (32.8)	18 (28.6)	
3	1655 (32.9)	219 (32.4)		152 (43.3)	22 (34.9)	
Education Level						
Incomplete primary	191 (3.8)	45 (6.7)	<0.001	19 (5.4)	1 (1.6)	0.005
Complete primary	257 (5.1)	48 (7.1)		11 (3.1)	7 (11.1)	
High school complete	1507 (29.9)	215 (31.8)		80 (22.7)	20 (31.7)	
University complete	3086 (61.2)	368 (54.4)		242 (68.8)	35 (55.6)	
Marital Status						
Married	3453 (70.3)	452 (66.9)	0.007	187 (53.1)	40 (63.5)	0.332
Separated/divorced	784 (15.6)	126 (18.6)		70 (19.9)	8 (12.7)	
Single	420 (8.3)	47 (7.0)		58 (16.5)	10 (15.9)	
Widower	139 (2.8)	16 (2.4)		18 (5.1)	1 (1.6)	
Other (with previous union)	155 (3.1)	35 (5.2)		18 (5.4)	4 (6.3)	
Ethnicity						
Not white	2069 (41.6)	302 (45.6)	0.047	139 (38.8)	32 (52.5)	0.065
White	2909 (58.4)	360 (54.4)		210 (60.2)	29 (47.5)	
lipid-lowering drugs						
No	4438 (88.0)	599 (88.6)	0.667	331 (94.0)	59 (93.7)	0.781 *
Yes	603 (12.0)	77 (11.4)		21 (6.0)	4 (6.3)	
Body mass index						
Thin	35 (0.7)	2 (0.3)	<0.001	7 (2.0)	4 (6.3)	0.051
Normal	1926 (38.2)	182 (26.9)		209 (59.4)	28 (44.4)	
Overweight	2088 (41.4)	323 (47.8)		98 (27.8)	24 (38.1)	
Obesity	990 (19.6)	169 (25.0)		38 (10.8)	7 (11.1)	
Physical activity in leisure						
Low	3627 (73.1)	517 (77.3)		229 (65.2)	45.0 (71.4)	0.12
Moderate	781 (15.7)	91 (13.6)	0.063	77 (21.9)	7.0 (11.1)	
High	557 (11.2)	61 (9.1)		45 (12.8)	11 (17.5)	
Smoking						
Never smoked	2679 (53.2)	202 (29.9)	<0.001	187 (53.1)	17 (27.0)	<0.001
**Ex-smoker**	**1604 (31.8)**	**278 (41.1)**		**122 (34.7)**	**28 (44.4)**	
**Smoker**	**757 (15.0)**	**196 (29.0)**		**43 (12.2)**	**18 (28.6)**	

Chi-square tests. Data were expressed (%) for categorical variables. * *p* < 0.05.

**Table 4:** Anthropometric, diet and biochemical variables dependent on HDL-C levels and alcohol consumption.

	Normal			HALP		
	Median ± SD		*p*	Median ± SD		*p*
	**Moderate**	**Excessive**		**Moderate**	**Excessive**	
	5041 (88.2)	676 (11.8)		352 (84.8)	63 (15.2)	
Age (years)	51.7 ± (9.0)	52.6 (8.2)	0.007	53.9 ± (8.9)	54.8 ± (7.8)	0.436
Weight (Kg)	74.6 ± (14.8)	79.0 ± (14.4)	<0.001	63.9 ± (10.9)	67.5 ± (13.6)	0.023
Height (Mts)	166.9 ± (9.2)	169.2 ± (8.6)	<0.001	161.5 ± (8.2)	164.6 ± (8.0)	0.006
BMI (Kg/m^2^)	26.9 ± (4.4)	27.5 ± (4.2)	<0.001	24.5 ± (3.8)	24.8 ± (4.4)	0.542
Waist circumference (cm)	91.3 ± (12.2)	95.9 ± (11.6)	<0.001	82.2 ± (10.3)	85.8 ± (11.2)	0.011
Kilocalories	2892.8 ± (981.6)	3233.9 ± (1064.6)	<0.001	2633.1 ± (867.8)	2885.3 ± (1041.1)	0.04
Lipids	93.3 ± (35.9)	102.0 ± (39.9)	<0.001	86.2 ± (34.0)	87.5 ± (39.0)	0.784
Carbohydrates	372.8 ± (143.4)	387.7 ± (152.1)	0.017	333.0 ± (122.7)	342.1 ± (140.9)	0.598
Proteins	136.5 ± (52.4)	147.1 ± (57.1)	<0.001	129.6 ± (50.9)	137.0 ± (62.7)	0.304
Wine (mL/week)	210.0 ± (293.5)	536.2 ± (862.1)	<0.001	243.9 ± (325.2)	507.8 ± (738.9)	0.007
Beer (mL/week)	972.6 ± (1029.8)	4425.4 ± (2663.5)	<0.001	759.0 ± (885.7)	4056.9 ± (2530.1)	<0.001
Spirits (mL/week)	22.6 ± (60.5)	166.7 ± (263.6)	<0.001	23.0 ± (69.7)	199.2 ± (393.9)	<0.001
Total alcohol (mL/week)	1205.3 ± (993.2)	5128.3 ± (2276.5)	0.017	1025.9 ± (854.4)	4764.0 ± (2258.0)	<0.001
Total alcohol (g/week)	66.1 ± (47.0)	281.0 ± (97.5)	<0.001	60.9 ± (44.4)	273.1 ± (144.5)	<0.001
TC (mg/dL)	217.9 ± (40.1)	226.4 ± (43.3)	<0.001	241.7 ± (40.1)	239.6 ± (41.7)	0.703
Triglycerides (mg/dL)	140.3 ± (102.4)	180.0 ± (148.9)	<0.001	90.8 ± (41.4)	109.4 ± (144.0)	0.313
HDL-C (mg/dL)	56.4 ± (10.6)	57.9 ± (11.0)	0.001	93.5 ± (10.9)	92.9 ± (13.5)	0.66
LDL-C (mg/dL)	134.2 ± (34.3)	134.9 ± (35.7)	0.586	130.3 ± (36.9)	125.5 ± (32.7)	0.339

*t*-test data were expressed as mean±SD for continuous variables. *p* < 0.05.

**Table 5:** Binary logistic regression (OR (95% CI)) between alcoholic beverage consumption and extremely high levels of HDL-C (HALP).

	OR Crude	OR Adjusted 1 *	OR Adjusted 2 **
	OR CI 95%	OR CI 95%	OR CI 95%
Moderate	1	1	1
Excessive	1.33 (1.1–1.7)	1.73 (1.2–2.3)	1.92 (1.4–2.5)

* Adjusted for: sex, age and income. ** Adjusted for:physical activity, kilocalories and BMI.

## Data Availability

The data presented in this study are available on request from the corresponding author.

## References

[1] World Health Organization (2018). Global Status Report on Alcohol and Health 2018: Executive Summary. https://apps.who.int/iris/rest/bitstreams/1217404/retrieve#:~:text=In2016%2C.

[2] Grucza R.A., Sher K.J., Kerr W.C., Krauss M.J., Lui C.K., McDowell Y.E., Hartz S., Virdi G., Bierut L.J. (2018). Trends in Adult Alcohol Use and Binge Drinking in the Early 21st-Century United States: A Meta-Analysis of 6 National Survey Series. Alcohol. Clin. Exp. Res..

[3] Manthey J., Shield K.D., Rylett M., Hasan O.S.M., Probst C., Rehm J. (2019). Global alcohol exposure between 1990 and 2017 and forecasts until 2030: A modelling study. Lancet.

[4] Jomard A., Osto E. (2020). High Density Lipoproteins: Metabolism, Function, and Therapeutic Potential. Front. Cardiovasc. Med..

[5] Julve J., Escolà-Gil J.C. (2022). High-Density Lipoproteins and Cardiovascular Disease: The Good, the Bad, and the Future II. Biomedicines.

[6] Rye K.-A., Barter P.J. (2014). Cardioprotective functions of HDLs. J. Lipid Res..

[7] Cordero A., Moreno-Arribas J., Bertomeu-González V., Agudo P., Miralles B., Masiá M.D., López-Palop R., Bertomeu-Martínez V. (2012). Low Levels of High-Density Lipoproteins Cholesterol Are Independently Associated With Acute Coronary Heart Disease in Patients Hospitalized for Chest Pain. Revista Española Cardiología.

[8] Brown B.G., Zhao X.-Q., Chait A., Fisher L.D., Cheung M.C., Morse J.S., Dowdy A.A., Marino E.K., Bolson E.L., Alaupovic P. (2001). Simvastatin and Niacin, Antioxidant Vitamins, or the Combination for the Prevention of Coronary Disease. N. Engl. J. Med..

[9] Besler C., Heinrich K., Rohrer L., Doerries C., Riwanto M., Shih D.M., Chroni A., Yonekawa K., Stein S., Schaefer N. (2011). Mechanisms underlying adverse effects of HDL on eNOS-activating pathways in patients with coronary artery disease. J. Clin. Investig..

[10] Patel P.J., Khera A.V., Wilensky R.L., Rader D.J. (2013). Anti-oxidative and cholesterol efflux capacities of high-density lipoprotein are reduced in ischaemic cardiomyopathy. Eur. J. Heart Fail..

[11] Hirano K.-I., Yamashita S., Nakajima N., Arai T., Maruyama T., Yoshida Y., Ishigami M., Sakai N., Kameda-Takemura K., Matsuzawa Y. (1997). Genetic Cholesteryl Ester Transfer Protein Deficiency Is Extremely Frequent in the Omagari Area of Japan. Marked hyperalphalipoproteinemia caused by CETP gene mutation is not associated with longevity. Arter. Thromb. Vasc. Biol..

[12] Yi S.-W., Park S.-J., Yi J.-J., Ohrr H., Kim H. (2020). High-density lipoprotein cholesterol and all-cause mortality by sex and age: A prospective cohort study among 15.8 million adults. Int. J. Epidemiol..

[13] Kontush A., de Faria E.C., Chantepie S., Chapman M.J. (2004). Antioxidative activity of HDL particle subspecies is impaired in hyperalphalipoproteinemia: Relevance of enzymatic and physicochemical properties. Arterioscler. Thromb. Vasc. Biol..

[14] Hirano K.-I., Nagasaka H., Kobayashi K., Yamaguchi S., Suzuki A., Toda T., Doyu M. (2014). Disease-associated marked hyperalphalipoproteinemia. Mol. Genet. Metab. Rep..

[15] Borggreve S.E., Hillege H.L., Wolffenbuttel B.H., De Jong P.E., Zuurman M.W., Van Der Steege G., Van Tol A., Dullaart R.P.F. (2006). An Increased Coronary Risk Is Paradoxically Associated with Common Cholesteryl Ester Transfer Protein Gene Variations That Relate to Higher High-Density Lipoprotein Cholesterol: A Population-Based Study. J. Clin. Endocrinol. Metab..

[16] Laurinavicius A.G., Santos I.S., Santos R.D., Bensenor I.M., Conceição R.D., Lotufo P.A. (2016). Extremely elevated HDL-cholesterol levels are not associated with increased carotid intima-media thickness: Data from ELSA Brasil. J. Clin. Lipidol..

[17] Curb J.D., Abbott R.D., Rodriguez B.L., Masaki K., Chen R., Sharp D.S., Tall A.R. (2004). A prospective study of HDL-C and cholesteryl ester transfer protein gene mutations and the risk of coronary heart disease in the elderly. J. Lipid Res..

[18] Hulley S.B., Cohen R., Widdowson G. (1977). Plasma High-Density Lipoprotein Cholesterol Level: Influence of Risk Factor Intervention. JAMA.

[19] Bagnardi V., Zatonski Scotti L., La Vecchia C., Corrao G. (2008). Does drinking pattern modify the effect of alcohol on the risk of coronary heart disease? Evidence from a meta-analysis. J. Epidemiol. Community Health.

[20] Millwood I.Y., Walters R.G., Mei X.W., Guo Y., Yang L., Bian Z., Bennett D.A., Chen Y., Dong C., Hu R. (2019). Conventional and genetic evidence on alcohol and vascular disease aetiology: A prospective study of 500,000 men and women in China. Lancet.

[21] So-Armah K.A., Cheng D.M., Freiberg M.S., Gnatienko N., Patts G., Ma Y., White L., Blokhina E., Lioznov D., Doyle M.F. (2019). Association between alcohol use and inflammatory biomarkers over time among younger adults with HIV—The Russia ARCH Observational Study. PLoS ONE.

[22] Castelli W.P., Doyle J.T., Gordon T., Hames C.G., Hjortland M.C., Hulley S.B., Kagan A., Zukel W.J. (1977). HDL cholesterol and other lipids in coronary heart disease. The cooperative lipoprotein phenotyping study. Circulation.

[23] van der Steeg W.A., Holme I., Boekholdt S.M., Larsen M.L., Lindahl C., Stroes E.S., Tikkanen M.J., Wareham N.J., Faergeman O., Olsson A.G. (2008). High-Density Lipoprotein Cholesterol, High-Density Lipoprotein Particle Size, and Apolipoprotein A-I: Significance for Cardiovascular Risk. The IDEAL and EPIC-Norfolk Studies. J. Am. Coll. Cardiol..

[24] Averina M., Nilssen O., Brenn T., Brox J., Arkhipovsky V.L., Kalinin A.G. (2005). Social and lifestyle determinants of depression, anxiety, sleeping disorders and self-evaluated quality of life in Russia—A population-based study in Arkhangelsk. Soc. Psychiatry Psychiatr. Epidemiol..

[25] O’Keefe J.H., Bybee K.A., Lavie C.J. (2007). Alcohol and Cardiovascular Health: The Razor-Sharp Double-Edged Sword. J. Am. Coll. Cardiol..

[26] Hansel B., Thomas F., Pannier B., Bean K., Kontush A., Chapman M.J., Guize L., Bruckert E. (2010). Relationship between alcohol intake, health and social status and cardiovascular risk factors in the Urban Paris-Ile-de-France Cohort: Is the cardioprotective action of alcohol a myth?. Eur. J. Clin. Nutr..

[27] Enríquez Martínez O.G., Luft V.C., Faria CP de Molina M.D.C.B. (2019). Alcohol consumption and lipid profile in participants of the Longitudinal Study of Adult Health (ELSA-BRASIL). Nutr. Hosp..

[28] Nova E., Mauro-Martín I.S., Díaz-Prieto L.E., Marcos A. (2019). Wine and beer within a moderate alcohol intake is associated with higher levels of HDL-c and adiponectin. Nutr. Res..

[29] Huang S., Li J., Shearer G.C., Lichtenstein A.H., Zheng X., Wu Y., Jin C., Wu S., Gao X. (2017). Longitudinal study of alcohol consumption and HDL concentrations: A community-based study. Am. J. Clin. Nutr..

[30] Lohman T.G., Roche A.F., Martorell R. (1988). Anthropometric standardization reference manual.

[31] Molina M.D.C.B., Benseñor I.M., Cardoso L.D.O., Velasquez-Melendez G., Drehmer M., Pereira T.S.S., Faria C.P.D., Melere C., Manato L., Gomes A.L.C. (2013). Reprodutibilidade e validaderelativa do Questionário de FrequênciaAlimentar do ELSA-Brasil Reproducibility and relative validity of the Food Frequency Questionnaire used in the ELSA-BrasilReproducibilidad y validezrelativa del Cuestionario de Frecuenc. Cadernos de SaúdePública.

[32] Martinez O.G.E., Aprelini C.M.D.O., Moreira T.K.B., Alves S.A., Pereira T.S.S., Siqueira J.H., Ferriani L.O., de Faria C.P., Molina M.D.C.B. (2021). Reproducibility and validity ELSA-Brasil Food Frequency Questionnaire. Revista Española Nutrición Humana y Dietética.

[33] Chor D., Alves M.G.D.M., Giatti L., Cade N.V., Nunes M.A., Molina M.D.C.B., Benseñor I.M., Aquino E.M., Passos V., Santos S.M. (2013). Questionnaire development in ELSA-Brasil: Challenges of a multidimensional instrument. Rev. SaúdePublica.

[34] U.S. Department of Health and Human Services Dietary Guidelines for Americans 2005. https://health.gov/sites/default/files/2020-01/DGA2005.pdf.

[35] Fedeli L.G., Vidigal P.G., Leite C.M., Castilhos C.D., Pimentel R.A., Maniero V.C., Mill J.G., A Lotufo P., Pereira A.C., Bensenor I.M. (2013). Logistica de coleta e transporte de material biologico e organizacao do laboratorio central no ELSA-Brasil. Revista SaúdePública.

[36] Cardoso L.G.V., Melo A.P.S., César C.C. (2015). Prevalência do consumomoderado e excessivo de álcool e fatoresassociados entre residentes de ComunidadesQuilombolas de Vitória da Conquista, Bahia, Brasil. Ciência&SaúdeColetiva.

[37] Oates C.P., Koenig D., Rhyne J., Bogush N., O’Connell J., Mitchell B.D., Miller M. (2018). Novel polymorphisms associated with hyperalphalipoproteinemia and apparent cardioprotection. J. Clin. Lipidol..

[38] El Khoudary S.R., Hutchins P.M., Matthews K.A., Brooks M.M., Orchard T.J., Ronsein G.E., Heinecke J.W. (2016). Cholesterol Efflux Capacity and Subclasses of HDL Particles in Healthy Women Transitioning Through Menopause. J. Clin. Endocrinol. Metab..

[39] Fan A.Z., Dwyer J.H. (2007). Sex differences in the relation of HDL cholesterol to progression of carotid intima-media thickness: The Los Angeles Atherosclerosis Study. Atherosclerosis.

[40] WHO (World Health Organization) (1998). Obesity: Preventing and Managing the Global Epidemic: Report of a Who Consultation on Obesity.

[41] Shohaimi S., Boekholdt M.S., Luben R., Wareham N.J., Khaw K.-T. (2014). Distribution of lipid parameters according to different socio-economic indicators- the EPIC-Norfolk prospective population study. BMC Public Health.

[42] Espírito Santo L.R., Faria T.O., Silva C.S.O., Xavier L.A., Reis V.C., Mota G.A., Silveira M.F., Mill J.G., Baldo M.P. (2019). Socioeconomic status and education level are associated with dyslipidemia in adults not taking lipid-lowering medication: A population-based study. Int. Health.

[43] Benetou V., Chloptsios Y., Zavitsanos X., Karalis D., Naska A., Trichopoulou A. (2000). Total cholesterol and HDL-cholesterol in relation to socioeconomic status in a sample of 11,645 Greek adults: The EPIC study in Greece. European Prospective Investigation into Nutrition and Cancer. Scand. J. Public Health.

[44] Lara M., Amigo H. (2018). Association between education and blood lipid levels as income increases over a decade: A cohort study. BMC Public Health.

[45] Wilkins J.T., Ning H., Stone N.J., Criqui M.H., Zhao L., Greenland P., Lloyd-Jones D.M. (2014). Coronary Heart Disease Risks Associated with High Levels of HDL Cholesterol. J. Am. Heart Assoc..

[46] Dai H., Alsalhe T.A., Chalghaf N., Riccò M., Bragazzi N.L., Wu J. (2020). The global burden of disease attributable to high body mass index in 195 countries and territories, 1990–2017: An analysis of the Global Burden of Disease Study. PLoS Med..

[47] Powell-Wiley T.M., Poirier P., Burke L.E., Després J.-P., Gordon-Larsen P., Lavie C.J., Lear S.A., Ndumele C.E., Neeland I.J., Sanders P. (2021). Obesity and Cardiovascular Disease: A Scientific Statement from the American Heart Association. Circulation.

[48] Bora K., Pathak M.S., Borah P., Das D. (2017). Association of decreased high-density lipoprotein cholesterol (HDL-C) with obesity and risk estimates for decreased HDL-C attributable to obesity: Preliminary findings from a hospital-based study in a city from Northeast India. J. Prim. Care Community Health.

[49] Mandai N., Akazawa K., Hara N., Ide Y., Ide K., Dazai U., Chishaki A., Chishaki H. (2015). Body Weight Reduction Results in Favorable Changes in Blood Pressure, Serum Lipids, and Blood Sugar in Middle-Aged Japanese Persons: A 5-Year Interval Observational Study of 26,824 Cases. Glob. J. Health Sci..

[50] Finelli C., Crispino P., Gioia S., La Sala N., D’amico L., La Grotta M., Miro O., Colarusso D. (2016). The improvement of large High-Density Lipoprotein (HDL) particle levels, and presumably HDL metabolism, depend on effects of low-carbohydrate diet and weight loss. EXCLI J..

[51] Ryan D.H., Yockey S.R. (2017). Weight Loss and Improvement in Comorbidity: Differences at 5%, 10%, 15%, and Over. Curr. Obes. Rep..

[52] Bazzano L.A., Gu D., Reynolds K., Chen J., Wu X., Chen C.-S., Duan X., Chen J., He J. (2009). Alcohol consumption and risk of coronary heart disease among Chinese men. Int. J. Cardiol..

[53] Polsky S., Akturk H.K. (2017). Alcohol Consumption, Diabetes Risk, and Cardiovascular Disease Within Diabetes. Curr. Diabetes Rep..

[54] Park H., Shin S.K., Joo I., Song D.S., Jang J.W., Park J.-W. (2020). Systematic Review with Meta-Analysis: Low-Level Alcohol Consumption and the Risk of Liver Cancer. Gut Liver.

[55] Ma H., Li X., Zhou T., Sun D., Shai I., Heianza Y., Rimm E.B., Manson J.E., Qi L. (2021). Alcohol Consumption Levels as Compared With Drinking Habits in Predicting All-Cause Mortality and Cause-Specific Mortality in Current Drinkers. Mayo Clin. Proc..

[56] Barter P.J., Caulfield M., Eriksson M., Grundy S.M., Kastelein J.J.P., Komajda M., Lopez-Sendon J., Mosca L., Tardif J.-C., Waters D.D. (2007). Effects of Torcetrapib in Patients at High Risk for Coronary Events. N. Engl. J. Med..

[57] Bots M.L., Visseren F.L., Evans G.W., Riley W.A., Revkin J.H., Tegeler C.H., Shear C.L., Duggan W.T., Vicari R.M., Grobbee D.E. (2007). Torcetrapib and carotid intima-media thickness in mixed dyslipidemia (RADIANCE 2 study): A randomized, double-blind trial. Lancet.

[58] Schaefer E.J., Asztalos B.F. (2006). Cholesteryl ester transfer protein inhibition, high-density lipoprotein metabolism and heart disease risk reduction. Curr. Opin. Lipidol..

[59] Swertfeger D.K., Rebholz S., Li H., Shah A.S., Davidson W.S., Lu L.J. (2018). Feasibility of a plasma bioassay to assess oxidative protection of low-density lipoproteins by high-density lipoproteins. J. Clin. Lipidol..

[60] Movva R., Rader D.J. (2008). Laboratory Assessment of HDL Heterogeneity and Function. Clin. Chem..

[61] Sacks F.M., Liang L., Furtado J.D., Cai T., Davidson W.S., He Z., McClelland R.L., Rimm E.B., Jensen M.K. (2020). Protein-Defined Subspecies of HDLs (High-Density Lipoproteins) and Differential Risk of Coronary Heart Disease in 4 Prospective Studies. Arter. Thromb. Vasc. Biol..

[62] Axley P.D., Richardson C.T., Singal A.K. (2019). Epidemiology of Alcohol Consumption and Societal Burden of Alcoholism and Alcoholic Liver Disease. Clin. Liver Dis..

[63] Brien S.E., Ronksley P.E., Turner B.J., Mukamal K.J., A Ghali W. (2011). Effect of alcohol consumption on biological markers associated with risk of coronary heart disease: Systematic review and meta-analysis of interventional studies. BMJ.

[64] De Oliveira e Silva E.R., Foster D., McGee Harper M., Seidman C.E., Smith J.D., Breslow J.L., Brinton E.A. (2000). Alcohol consumption raises HDL cholesterol levels by increasing the transport rate of apolipoproteins A-I and A-II. Circulation.

